# Supplementary material for: Value of endoscopic ultrasonography in the observation of the remnant pancreas after pancreatectomy
Source: PLoS One. 2021 Jan 19;16(1):e0245447. doi: 10.1371/journal.pone.0245447 (PMC7815110; doi:10.1371/journal.pone.0245447)
Supplement: Attachment — Submitted filename: Response to Reviewer.docx [file pone.0245447.s002.docx]

**Nov. 27^th^. 2020**

**Joerg Heber**

**Editor-in-Chief**

**Associate Editors**

***PLOS ONE***

Dear Editor

We appreciate the opportunity to submit a revised version of our manuscript entitled, “**Value of endoscopic ultrasonography in the observation of the remnant pancreas after pancreatectomy**” (manuscript ID: PONE-D-20-25624) for consideration for publication in *PLOS ONE*.

We believe that we have addressed all concerns raised by the reviewers as detailed in the accompanying point-by-point responses.

All authors concur with the submission of this manuscript. We ascertain that none of the data in this manuscript have been previously reported, nor is the manuscript under consideration for publication elsewhere.

We hope that *PLOS ONE* now finds our manuscript suitable for publication. We appreciate your consideration of our work.

Sincerely Yours,

Hirotsugu Maruyama

Hirotsugu Maruyama, M.D., PhD.

Department of Gastroenterology

Osaka City University Graduate School of Medicine

1-4-3, Asahimachi, Abeno-ku, Osaka-City, Osaka, 545-8585, Japan

e-mail to; hiromaruyama99@gmail.com

Phone: +81-6-6645-3811

FAX: +81-6-6645-3813

**Point-by-Point Responses (PONE-D-20-25624)**

**Responses to the Editorial and Reviewer comments:**

We appreciate the editor’s positive and helpful comments about our paper. The reviewer raised some important points for improvement that we have now addressed, as summarized below. Please note that all changes are yellow highlights in the revised manuscript.

**Comment 1:**
- the Authors should state if PC patients received EUS before Surgery and if data were compared with those achieved during the EUS performed on RP.

**Response 1:**

Thank you for your constructive comment. We could not compare preoperative and postoperative EUS, because of several limitations. First, 32 patients received pre- and post-operative EUS; however, 13 patients did not receive preoperative EUS. Second, it is difficult to compare pre- and post-operative EUS because all patients have been several years since preoperative EUS and the condition of the background pancreas has changed.

We described it in the DISCUSSION (limitation): " Our study has several limitations. First, the analyses were based on retrospectively collected data. Second, the variability in echoendoscopes and processors may change the capability to detect pancreatic lesions. Third, we did not compare pre-operative and post-operative EUS because, 13 patients did not receive pre-operative EUS and all patients had passed several years since pre-operative EUS. Therefore, the condition of the background pancreas had changed and accurate comparisons were difficult." (from page 22, line 4 to 7)

**Comment 2:**
- did the AUthors performe pancreatic margin frozen section examination? If they did, they shoud state at least how many IPMN have been found in the 45 cases object of the study.

**Response 2:**

Thank you for your constructive comment. All cases underwent pancreatic margin frozen section examination. Among the 45 cases, 14 were IPMN cases. In the text, we described "Thirty-third patients had PC. The other patients were 10 cases of IPMN and 2 cases of Intraductal Papillary Mucinous Carcinoma (IPMC)". Thus, 12 cases were described in the text. However, among the 33 cases of pancreatic cancer, 2 cases were pancreatic ductal adenocarcinoma concomitant with IPMN of the pancreas.

We described in the Results (Baseline characteristics of patients): " Thirty-three patients had PC, two of them had PC concomitant with IPMN of the pancreas. The other patients were 10 cases of IPMN and 2 cases of Intraductal Papillary Mucinous Carcinoma (IPMC)." (from page 12, line 8 to 11)

**Comment 3:**
- text and English Language should be revised.

**Response 3:**

We appreciate your comment.

Our manuscript had undergone English proofreading by a native speaker, and has been revised once again by a professional English language editing service; the calibration certificate has been attached. Please see the revised manuscript.
